# Ultrahigh-resolution full-color perovskite nanocrystal patterning for ultrathin skin-attachable displays

**DOI:** 10.1126/sciadv.add0697

**Published:** 2022-10-26

**Authors:** Jong Ik Kwon, Gyuri Park, Gwang Heon Lee, Jae Hong Jang, Nak Jun Sung, Seo Young Kim, Jisu Yoo, Kyunghoon Lee, Hyeonjong Ma, Minji Karl, Tae Joo Shin, Myoung Hoon Song, Jiwoong Yang, Moon Kee Choi

**Affiliations:** ^1^ Department of Materials Science and Engineering, Ulsan National Institute of Science and Technology (UNIST), Ulsan 44919, Republic of Korea.; ^2^ Department of Energy Science and Engineering, Daegu Gyeongbuk Institute of Science and Technology (DGIST), Daegu 42988, Republic of Korea.; ^3^ Graduate School of Semiconductor Materials and Devices Engineering, Center for Future Semiconductor Technology (FUST), Ulsan National Institute of Science and Technology (UNIST), Ulsan 44919, Republic of Korea.; ^4^ UNIST Central Research Facilities, Ulsan National Institute of Science and Technology, Ulsan 44919, Republic of Korea.; ^5^ Energy Science and Engineering Research Center, Daegu Gyeongbuk Institute of Science and Technology (DGIST), Daegu 42988, Republic of Korea.; ^6^ Center for Nanoparticle Research, Institute for Basic Science (IBS), Seoul 08826, Republic of Korea.

## Abstract

High-definition red/green/blue (RGB) pixels and deformable form factors are essential for the next-generation advanced displays. Here, we present ultrahigh-resolution full-color perovskite nanocrystal (PeNC) patterning for ultrathin wearable displays. Double-layer transfer printing of the PeNC and organic charge transport layers is developed, which prevents internal cracking of the PeNC film during the transfer printing process. This results in RGB pixelated PeNC patterns of 2550 pixels per inch (PPI) and monochromic patterns of 33,000 line pairs per inch with 100% transfer yield. The perovskite light-emitting diodes (PeLEDs) with transfer-printed active layers exhibit outstanding electroluminescence characteristics with remarkable external quantum efficiencies (15.3, 14.8, and 2.5% for red, green, and blue, respectively), which are high compared to the printed PeLEDs reported to date. Furthermore, double-layer transfer printing enables the fabrication of ultrathin multicolor PeLEDs that can operate on curvilinear surfaces, including human skin, under various mechanical deformations. These results highlight that PeLEDs are promising for high-definition full-color wearable displays.

## Teaser

Double-layer transfer printing enables ultrahigh-resolution full-color perovskite patterns for ultrathin skin-attachable displays.

## INTRODUCTION

Wearable displays with ultrahigh definition have been in great demand with the advancement of wearables, mobiles, and the Internet of Things (IoT) in recent years. Ideally, these displays must be mechanically deformable so that they can be conformally attached to various curvilinear surfaces to enhance wearability, even during active motion (*1*). Technological advances [i.e., ultrathin designs (*2*–*4*), stretchable interconnection designs (*5*), and intrinsically stretchable materials (*6*–*8*)] to achieve deformable form factors enable mechanical changes such as bending, rolling, and twisting. In addition, it is critical to design wearable displays with high efficiency, because the power supply to wearable electronics is limited (*9*). Furthermore, high-definition red/green/blue (RGB) subpixels are essential for visualizing various types of information on the limited screen sizes of wearable displays that can be worn on wrists, fingers, or eyes (*10*). For example, on a two-inch smartwatch display, a resolution of ~2200 pixels per inch (PPI) is required to express the resolution of a commercial 4K TV (3840 × 2160 pixels). Despite recent progress in display technology, the development of high-efficiency high-definition full-color wearable light-emitting diodes (LEDs) remains a challenging goal (*11*, *12*).

Metal halide perovskites exhibit considerable potential in optoelectronic applications because of their broadly tunable emissions with high brightness and color purity [full width at half maximum (FWHM) < 20 nm], high photoluminescence (PL) quantum yield (up to 100%), and low-cost solution-based fabrication (*13*–*21*). Moreover, the ultrathin thickness of perovskite light-emitting diodes (PeLEDs) (<1 μm, excluding the thickness of the substrate) makes them promising candidates for application to ultrathin and deformable (i.e., flexible, foldable, and stretchable) displays (*22*). With recent advances in synthesis (*23*–*25*), photophysics (*26*), and device engineering (*27*, *28*), the external quantum efficiencies (EQEs) of green- and red-emitting PeLEDs have reached approximately 23.4% (*29*) and 21.3% (*30*), respectively, which approach the theoretical limits. Most previous studies have been focused on improving the device performance of PeLEDs with monochromatic perovskite nanocrystal (PeNC) films fabricated by a spin-coating method (*23*–*30*). However, manufacturing high-definition full-color displays on the commercial scale poses several challenges, such as the development of patterning processes for RGB subpixels that are compatible with the operation of electroluminescent (EL) devices (*31*, *32*).

Conventional patterning processes (e.g., photolithography and inkjet printing) are unsuitable for the fabrication of highly efficient PeLEDs. During the photolithography process, perovskite materials can be easily degraded by polar solvents, moisture, and ultraviolet (UV) light, owing to their ionic bonding nature (*33*, *34*). In addition, the wet chemicals used in photolithography inevitably damage the underlying charge transport layers and/or PeNCs, thereby hindering the realization of high-performance full-color PeLEDs (*35*, *36*). Inkjet printing has also been widely studied as it enables simple processing through additive patterning (*37*, *38*); however, the additives (i.e., polymer matrix, surfactant, and viscosity modifier) used for uniform ink ejection and difficulty in forming ultrathin pixels (on the scale of tens of nanometers) suppress the EL properties of PeLEDs. Dry transfer printing (*39*–*41*) using a viscoelastic stamp can be a strategic option for fabricating high-definition PeNC pixels for EL devices. This process does not use wet chemicals, avoiding the solvent orthogonality issue and preventing cross-contamination with different colored pixels (*42*). This approach, however, has rarely been applied to PeNCs. The weak interaction energy between the PeNCs easily causes internal cracking of the thin film during the transfer process.

**Fig. 1.**
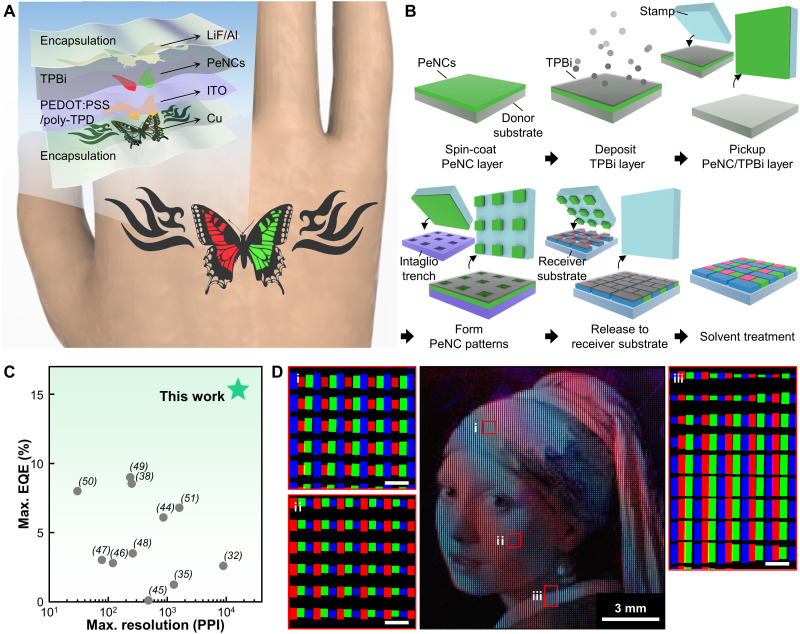
Development of the PeNC transfer printing. (**A**) Schematic illustration of a multicolor skin-attachable PeLED. (**B**) Illustration of the double-layer transfer printing process with RGB pixelated arrays of PeNCs. (**C**) Comparison of key characteristics (maximum resolution and maximum EQE) of the printed perovskites. The maximum resolution was determined by the smallest dot patterns of each work. (**D**) PL images of transfer-printed PeNC RGB pixelated patterns displaying the *Girl with a Pearl Earring* painting by J. Vermeer. Insets (i) to (iii) are magnified fluorescent images showing scaled RGB subpixels to express the full-color image. Scale bars, 100 μm.

Here, we present high-resolution full-color PeNC patterns printed via double-layer dry transfer printing for ultrathin skin-attachable PeLED displays (Fig. 1A). Notably, the introduction of an organic charge transport layer between the metal halide PeNCs and poly(dimethylsiloxane) (PDMS) stamp prevents internal cracking of the PeNC film during the transfer printing process. This technique achieves uniform high-definition PeNC patterns of the desired size and readily produces submicrometer patterns. Furthermore, the dimension of the aligned RGB subpixels can be reduced to 3 μm by 3 μm, suggesting that double-layer transfer printing can be used to create full-color high-definition displays. These printed PeNC layers exhibit excellent optical and electrical properties in the EL devices, with EQEs of 15.3, 14.8, and 2.5% for red, green, and blue PeLEDs, respectively. Last, the ultrathin PeLEDs can be applied as freely deformable LEDs that can withstand various mechanical deformations such as bending, folding, wrinkling, and twisting.

## RESULTS

### High-definition RGB pixels using double-layer transfer printing

Double-layer transfer printing for PeNCs was developed to produce precise RGB pixels for high-resolution full-color displays (Fig. 1). Red, green, and blue CsPbX_3_ (X = Cl, Br, and I) PeNCs with halide-ion-pair ligands (*25*) were used because of their outstanding optical characteristics and enhanced chemical stability. Their synthetic procedures and characteristics are described in Materials and Methods and the Supplementary Materials (figs. S1 to S8). The procedure for double-layer transfer printing is illustrated in Fig. 1B: The PeNC layer is spin-cast on a self-assembled monolayer-treated donor substrate, followed by thermal evaporation of 2,2′,2′′-(1,3,5-benzinetriyl)-tris(1-phenyl-1-*H*-benzimidazole) (TPBi). This PeNC/TPBi double layer is picked up swiftly with a viscoelastomeric PDMS stamp (pickup step) and gently contacted onto the intaglio trench (receiver substrate with reverse patterns; formation of PeNC patterns). Owing to the significant difference in surface energy between the PDMS stamp (19.8 mJ m^−2^) and intaglio trench (>200 mJ m^−2^), the double layer in contact with the intaglio trench is selectively removed, and the remaining part over the stamp (desired pattern) is transferred to the receiver substrate (release step) (*41*). By repeating this process for PeNCs of different colors, patterns with RGB subpixels can be created, indicating that this printing method can be applied to ultrahigh-resolution full-color displays. Solvent treatment is conducted to enhance the junction property between the emitting and charge transport layers for further LED applications. As shown in Fig. 1C, the double-layer transfer method not only produces patterns with higher resolution but also is applicable to the printing of PeLEDs with superior efficiencies, compared to the reported patterning processes for perovskite materials in the representative literature (fig. S9 and table S1) (*32*, *35*, *38*, *43*–*51*). High-resolution PL images, such as *Girl with a Pearl Earring* by J. Vermeer (Fig. 1D) and *The Scream* by E. Munch (fig. S10), can be displayed using double-layer transfer printing. As shown in the insets of Fig. 1D, the color of each pixel can be expressed by scaling the RGB subpixels.

**Fig. 2.**
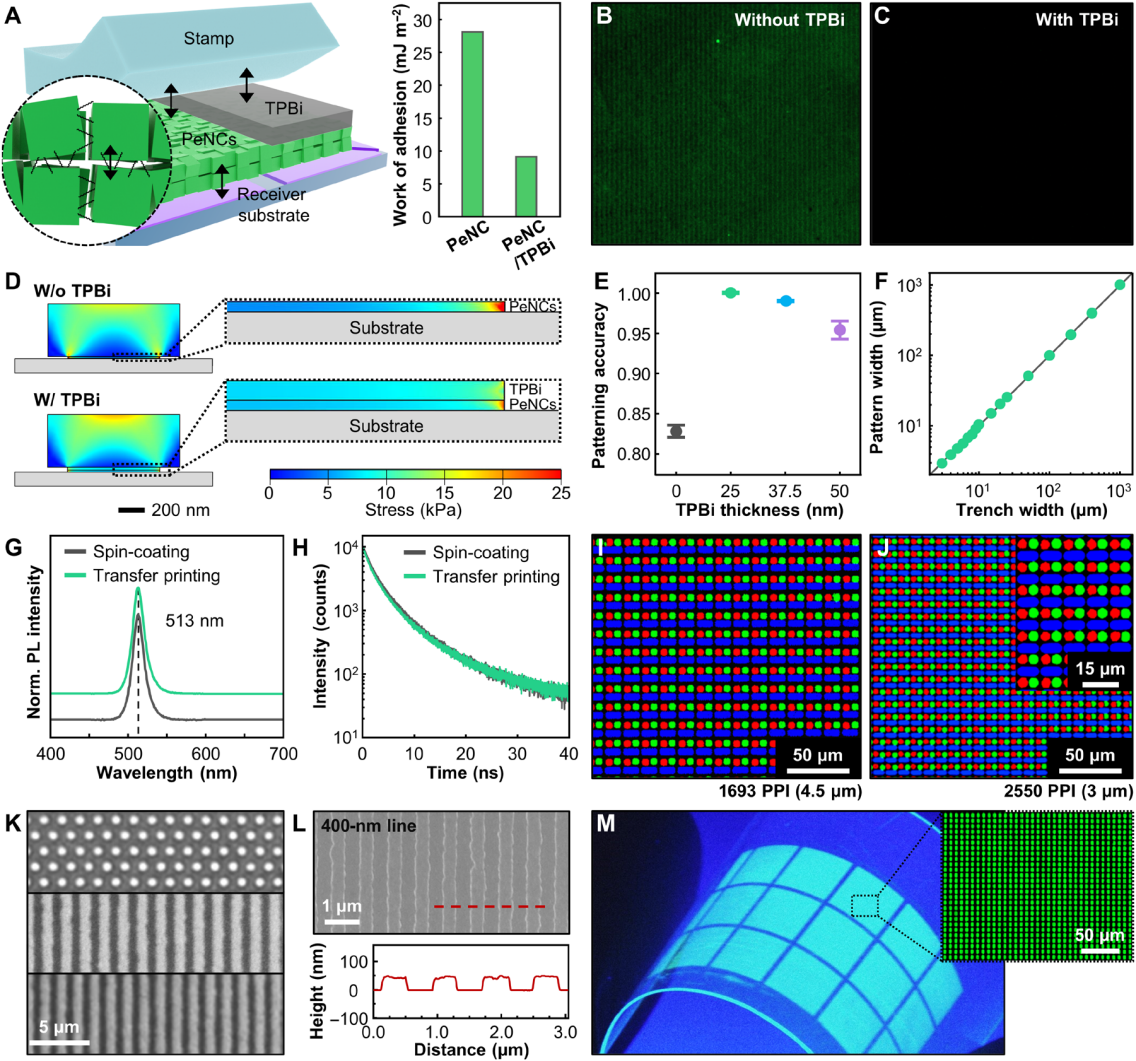
Ultrahigh-resolution, double-layer transfer printing of PeNCs. (**A**) Schematic illustration of the release step with the PeNC/TPBi double layer. The graph compares the work of adhesion between the PDMS stamp and PeNC and PeNC/TPBi layers. (**B** and **C**) Fluorescence microscopic images showing PeNC residues on the stamps after the release step (B) without and (C) with the TPBi layer. (**D**) FEM simulations performed to estimate the strain distribution in the PeNC layers with (bottom) and without (top) an organic layer during the release step in the double-layer transfer printing process. (**E**) Statistical analysis of the patterning accuracy at different TPBi thicknesses. (**F**) Statistical analysis of the achieved PeNC pattern width compared to the trench width with a 25-nm-thick TPBi layer. (**G**) PL spectra and (**H**) time-resolved PL decay curves of the spin-coated and transfer-printed PeNC layers. (**I** and **J**) Fluorescence microscopic images of the pixelated RGB PeNC patterns with (I) 1693 and (J) 2550 PPI resolutions. (**K**) Optical images of the submicrometer PeNC patterns: 800-nm dot (top), 800-nm line (middle), and 600-nm line (bottom). (**L**) Scanning electron microscopy image with 400-nm PeNC line patterns. The inset depicts the atomic force microscope topography results for the 400-nm line patterns. (**M**) PL image of the large-area transfer-printed PeNC layer under UV irradiation (λ = 365 nm). Inset shows a magnified fluorescence microscopic image of the pixelated PeNCs.

We systematically studied how double-layer transfer printing can successfully create patterns with fine RGB subpixels, which cannot be achieved using conventional transfer printing. Figure 2 (A to D) depicts the release of the PeNC/TPBi double layer from the stamp to the receiver substrate. In the release step, the picked-up PeNC layer on the PDMS stamp must be completely transferred to the receiver substrate at a low peeling rate according to the difference in the separation energy. The critical peeling rate (*G*) is expressed by Eq. 1 (*39*)

$${G^{\text{PeNCs}/\text{stamp}}}_{\text{crit}.}(v)<{G^{\text{PeNCs}/\text{receiver substrate}}}_{\text{crit}.}$$ (1)

Without additional organic layers (i.e., conventional transfer printing), numerous PeNCs still stick onto the surface of the stamp even after the release step, because of internal cracking of the PeNC layer (Fig. 2B). Consequently, the partially transferred PeNC layer exhibits an irregular shape, thickness, and fluorescence (fig. S11). This failure can be attributed to the weak van der Waals interactions between the PeNCs. The ligand density on the metal halide PeNC surface (1.7 nm^−1^) (*52*) is much lower than that of conventional CdSe quantum dots with long carbon chains (3.1 nm^−1^) (*53*), which are the most widely used materials for studying the patterning of nanocrystals. In addition, the use of short-chain ligands is preferred for PeNCs to improve charge transport within the PeLEDs (*54*), thereby weakening the van der Waals interactions (*55*). Therefore, these weak interactions between the PeNCs cause mutual delamination within the PeNC layer and exfoliation of some PeNCs to the viscoelastic PDMS stamp during the release process.

The introduction of an organic layer (TPBi) prevents mutual delamination of the PeNCs during transfer printing (Fig. 2C and fig. S11). The low work of adhesion between the PDMS stamp and TPBi layer enables easy delamination of the PeNC/TPBi double layer from the stamp, without any damage to the emission layer (Fig. 2A and table S2). Other organic materials can be used instead of TPBi (fig. S12). In addition, as shown in the finite element method (FEM) simulation at the release step, the stress distribution on the PeNC layer becomes uniform with the organic layer, preventing the internal cracking of the PeNC layer (Fig. 2D and Materials and Methods). The patterning yield can reach 100% with the optimum thickness of the organic layer (~25 nm; Fig. 2E and fig. S13). Excessive thickness of the organic layer (~50 nm) hinders the acquisition of fine PeNC patterns, which can be attributed to slipping and internal cracking inside the organic layer. As shown in Fig. 2F, the shapes and sizes of the transfer-printed PeNC patterns precisely match the designed patterns, regardless of the pattern size (from 3 μm to 1 mm; figs. S14 to S16). Moreover, the PeNC patterns can be perfectly printed onto various charge transport layers required for PeLED fabrication (fig. S17). Furthermore, the developed double-layer transfer printing does not cause any physical or chemical damage to the PeNC layer. The emission wavelength (513 nm) and exciton lifetime of the transfer-printed PeNCs are almost identical to those of the spin-coated PeNCs (Fig. 2, G and H, and figs. S18 and S19).

Using sequential transfer printing of PeNC patterns with different colors, microscale RGB PeNC subpixels can be aligned for full-color display applications with a transfer yield of ~100%. Figure 2 (I and J) and fig. S20 show RGB subpixel arrays, from 847 PPI (subpixel size, 12 μm) to 2550 PPI (subpixel size, 3 μm), without defects and cross-contamination between subpixels of different colors. Moreover, the pixel size is reduced to the nanometer scale for single-color PeNC patterns (Fig. 2K). The smallest feature size of the monochromatic patterns is ~400 nm (corresponding to 33,000 line pairs per inch), which is close to the optical diffraction limit (Fig. 2L) (*56*). Furthermore, the developed double-layer transfer printing offers large-area transfer printing, which is essential for mass manufacturing. Figure 2M shows the large-area transfer printing of hierarchical 3-μm-width dot arrays with an area of 630 mm^2^ on a flexible substrate.

### Double-layer transfer-printed PeLEDs

**Fig. 3.**
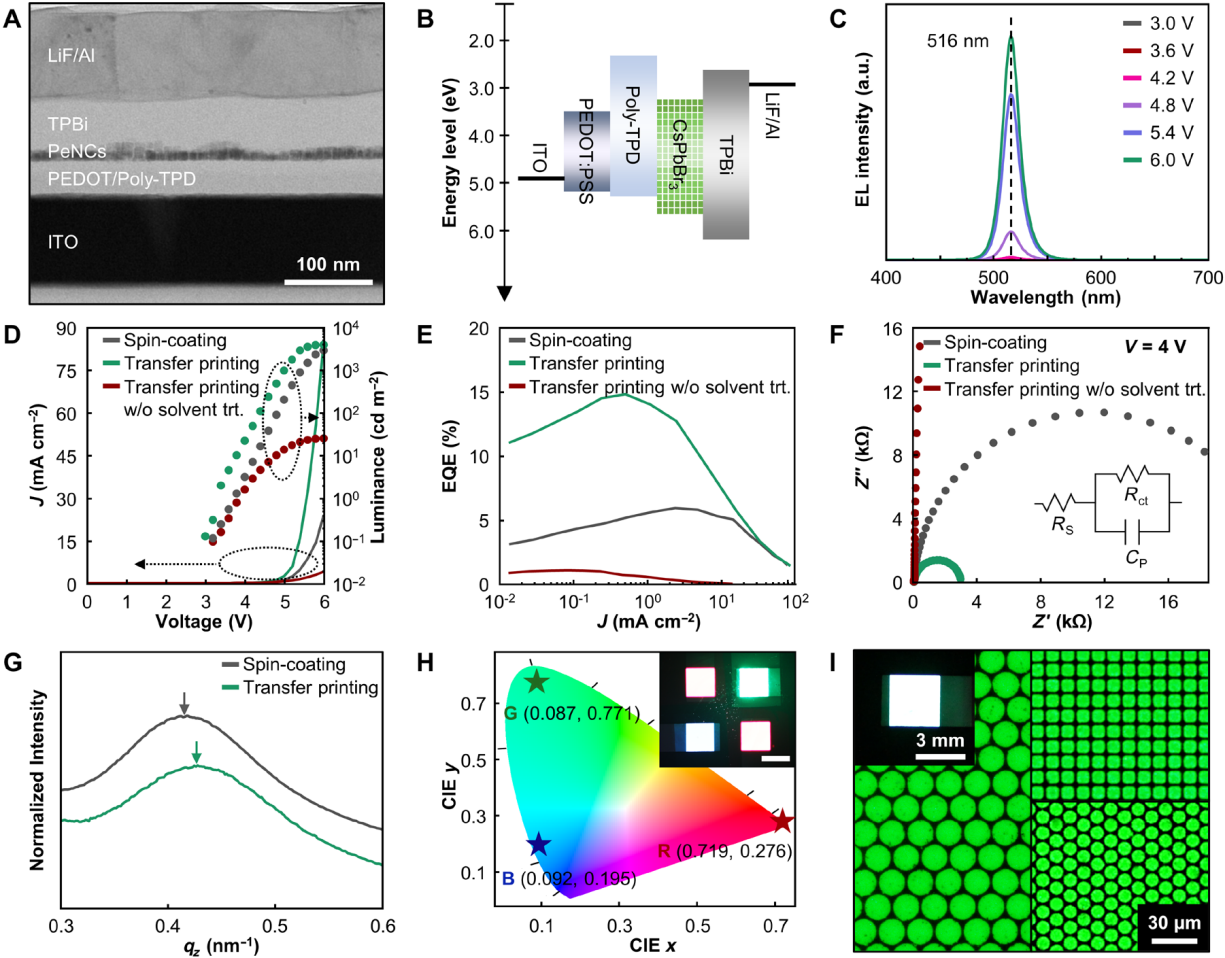
EL characteristics of transfer-printed PeLEDs. (**A**) Cross-sectional TEM image and (**B**) energy band diagram of the transfer-printed PeLEDs. (**C**) EL spectra of the transfer-printed green PeLEDs at various applied biases. (**D**) *J-V-L* curves and (**E**) EQE versus current density of the green PeLEDs obtained using transfer printing, transfer printing without solvent treatment, and spin-coating of the PeNC layers. (**F**) Electrochemical impedance analysis results. Inset shows the equivalent circuit model. (**G**) Normalized GI-SAXS data in the out-of-plane direction with different PeNC layer–forming processes. (**H**) CIE 1931 *xy* chromaticity diagram of the transfer-printed RGB PeLEDs. Inset shows EL image revealing transfer-printed RGB multicolor PeLEDs at 4.0 V. Scale bar, 3 mm. (**I**) Optical microscopy images of the EL emission from high-resolution transfer-printed green PeLEDs. Inset shows the photograph of the transfer-printed PeLED with green PeNC circle arrays with a diameter of 16 μm.

Double-layer transfer printing was applied to fabricate PeLEDs (see Materials and Methods for the detailed fabrication process). A cross-sectional transmission electron microscopy (TEM) image and an energy band diagram of the transfer-printed green PeLEDs are shown in Fig. 3 (A and B), respectively. For effective carrier injection from the electrodes, poly(*N*,*N*′-*bis*-4-butylphenyl-*N*,*N*′-bisphenyl)benzidine (poly-TPD) and TPBi were adopted as the hole transport layer (HTL) and electron transport layer, respectively. The PeNC (~13 nm)/TPBi (~25 nm) double layer was then transfer-printed onto the spin-cast poly-TPD layer without interference on the underlying charge transport layers. Solvent treatment with methyl acetate was performed to enhance the interface characteristics between the emitter and charge transport layers, as discussed below. Next, a 50-nm-thick TPBi layer, 1.5-nm-thick LiF, and 90-nm-thick Al electrode were sequentially deposited using a thermal evaporator. The PeLEDs with transfer-printed PeNCs exhibited sharp EL (FWHM ~18 nm) with a consistent peak position (516 nm), regardless of the applied bias (Fig. 3C).

Notably, our best device exhibits remarkable EL characteristics (peak EQE = 14.8%, peak current efficiency = 50.5 cd A^−1^, maximum brightness = 4159.3 cd m^−2^, and turn-on voltage < 3.0 V), which are superior to those of the reference device with a spin-coated PeNC layer (Fig. 3, D and E). To the best of our knowledge, the EQE of our transfer-printed PeLEDs is higher than that of printed PeLEDs in the previous works, regardless of the printing methods (e.g., photolithography and inkjet printing; Fig. 1C and table S1). The reproducibility of the transfer-printed PeLEDs was confirmed, as shown in the histograms for 20 devices (fig. S21). The solvent treatment reduced the turn-on voltage and enhanced the EQE of the transfer-printed PeLEDs, suggesting improved charge injection into the PeNC layer.

We systematically investigated the effects of transfer printing on the electrical characteristics of the PeLEDs. Electrochemical impedance analysis proved that the PeLEDs with the transfer-printed PeNCs exhibit low internal resistance (Fig. 3F) (*57*). The series resistance (*R*_s_) and charge-transport resistance (*R*_ct_) were obtained using the equivalent circuit model (table S3). At an applied bias of 4.0 V, the transfer-printed PeLEDs showed eightfold lower *R*_ct_ than that observed for the spin-coated PeLEDs. To verify the effects of the carrier injection, the electron-only device [EOD; indium tin oxide (ITO)/SnO_2_/PeNCs/TPBi/LiF/Al] and hole-only device [HOD; ITO/poly(3,4-ethylenedioxythiophene):poly(styrenesulfonate) (PEDOT:PSS)/poly-TPD/PeNCs/4,4′-bis(*N*-carbazolyl)biphenyl (CBP)/MoO*_x_*/Au] were fabricated with different PeNC film formation methods (fig. S22) (*32*, *50*). As shown in fig. S22A, the transfer-printed PeLEDs showed higher electron mobility than that of the spin-coated PeLEDs in the child region (*J* ∝ V*^n^*, *n* = 2), with improved current density without leakage current in the ohmic region (*J* ∝ V*^n^*, *n* = 1). This was attributed to the close packing of the transfer-printed PeNC layer. In the HOD, the transfer-printed PeLEDs exhibited significantly reduced injected hole leakage in the ohmic region (fig. S22B). The solvent treatment during the transfer printing procedure effectively decreased the internal resistance by modulating the interface between the HTL and PeNCs, deriving an efficient radiative recombination based on enhanced carrier injection.

Grazing incidence small-angle x-ray scattering (GI-SAXS) analysis was performed to confirm the interparticle distance and packing density in accordance with the film formation methods (Fig. 3G, fig. S23, and table S4). The interparticle distance of the transfer-printed PeNCs was reduced compared to that of the spin-coated PeNCs, owing to the pressure applied during transfer printing (*40*). The outstanding EL characteristics of the transfer-printed PeLEDs can be attributed to the reduced gaps in the transfer printed PeNCs. The device lifetime of the transfer-printed PeLED (22 min) was also longer than that of the spin-coated one (12 min) (fig. S24).

We applied the double-layer transfer printing to fabricate PeLEDs with other colors, such as red and blue. Figure 3H shows a photograph of the transfer-printed PeLEDs with vivid RGB emissions and their Commission Internationale de l’Eclairage (CIE) coordinates. The *J-V-L*, EQE, and EL spectra of transfer-printed red and blue PeLEDs are described in fig. S25, showing enhanced EL properties (peak EQE = 15.3 and 2.5% for red and blue PeLEDs, respectively) by the transfer printing process. The luminance and current efficiency of RGB PeLEDs are also summarized in fig. S26. Furthermore, high-resolution transfer-printed PeLEDs up to 2550 PPI were also successfully manufactured, proving the fidelity of our strategy (Fig. 3I and fig. S27). These findings suggest that the double-layer transfer printing is promising for full-color high-definition displays based on PeLEDs.

### Ultrathin skin-attachable displays based on transfer-printed PeLEDs

**Fig. 4.**
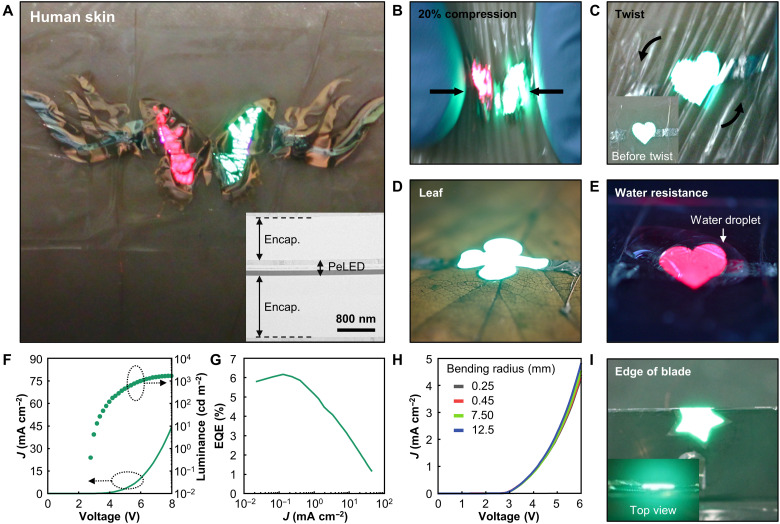
Ultrathin skin-attachable PeLEDs. (**A**) Photograph of ultrathin multicolor PeLEDs attached to human skin. Inset shows the cross-sectional TEM image of a skin-attachable transfer-printed PeLED (total thickness ~2.6 μm). (**B** and **C**) Ultrathin PeLEDs attached to human skin under various deformations: (B) 20% compression and (C) twist. Inset of (C) shows the skin-attached ultrathin PeLED before twist. (**D**) Ultrathin PeLEDs laminated on a leaf. (**E**) Demonstration of the water resistance of ultrathin PeLEDs. (**F** and **G**) Device characteristics of skin-attachable PeLEDs; (F) *J-V-L* characteristics and (G) EQE versus current density. (**H**) *J-V* characteristics of the skin-attachable PeLEDs under various strains (bending curvatures from 0.25 to 12.5 mm). (**I**) Photograph of an ultrathin PeLED folded on a razor blade edge (radius of curvature ~250 μm). Inset shows the top view of the folded PeLED.

The ultrathin form factor of the PeLEDs (~300 nm excluding the encapsulation layers) is suitable for the fabrication of wearable multicolor displays that can be conformally attached to human skin (Fig. 4A), considering that the induced strain during bending is proportional to the thickness of the bent materials (*2*). Using our developed double-layer transfer printing process, PeLEDs were successfully fabricated between a 1.3-μm-thick perylene/epoxy encapsulation substrate and a 1.0-μm-thick upper parylene encapsulation layer, as shown in the cross-sectional TEM image (Fig. 4A, inset). In this process, the rigid ITO electrode is placed on the neutral mechanical plane of the whole device structure, resulting in low induced strain on the PeLEDs upon mechanical deformation, without luminance roll-off (Fig. 4, A to E) (*58*). The ultrathin form of the PeLEDs enables conformal contact on various surface topologies, including human skin, a leaf, and the edge of a blade. As shown in Fig. 4 (B and C), the ultrathin PeLEDs are conformally attached to the human skin and maintain stable luminance under deformations including 20% compression and twist. Moreover, the parylene used for the upper and lower encapsulation is waterproof against moisture in air, sweat, and water droplets (Fig. 4E). Figure 4 (F and G) depicts the EL properties of skin-attachable PeLEDs with transfer-printed PeNC layers, revealing low turn-on voltages (<3.0 V) and high EQEs (~6.2%). These ultrathin PeLEDs demonstrate mechanical and electrical reliability even under various bending radii ranging from 250 μm to 12.5 mm (Fig. 4H). Last, Fig. 4I shows the stable light emission of ultrathin PeLEDs folded on the edges of razor blades (bending radius ~250 μm).

## DISCUSSION

In summary, we demonstrated high-definition RGB PeNC pixel arrays using double-layer dry transfer printing for ultrathin wearable PeLEDs. By applying a 25-nm-thick organic layer and solvent treatment, we could overcome the weak interparticle interaction of the metal halide PeNCs and achieve almost 100% transfer yield for patterns with various shapes and sizes. Furthermore, we could fabricate 400-nm-width monochromatic line patterns and a 2550 PPI RGB pixel array without cross-contamination among the RGB color alignment procedures. The mechanical pressure applied in the transfer printing process compactly packed the PeNCs and improved the EL performance of the transferred PeLED, yielding excellent EQEs (15.3, 14.8, and 2.5% for red, green, and blue, respectively). Consequently, ultrathin skin-attachable PeLEDs with enhanced optical (peak EQE ~6.2%) and mechanical (bending radius ~250 μm) characteristics were fabricated. We envision that our multicolor PeLEDs with ultrathin form factors will pave the way for practical applications of perovskite displays and wearable devices for IoT, virtual reality, and augmented reality devices in the future.

## MATERIALS AND METHODS

### Materials

Cesium carbonate (Cs_2_CO_3_; 99.9%), lead(II) iodide (PbI_2_; 99.999%), oleylamine (OAm; 70%), oleic acid (OA; 90%), 1-octadecene (1-ODE; 90%), hexane (anhydrous; 95%), methyl acetate (anhydrous; 99.5%), acetonitrile (anhydrous; 99.8%), and toluene (anhydrous; 99.8%) were purchased from Sigma-Aldrich. Lead(II) bromide (PbBr_2_; 99,998%), octadecyltrichlorosilane (ODTS; 95%), and SnO_2_ (15% in H_2_O colloidal dispersion) were purchased from Alfa Aesar. Didodecyldimethyl ammonium bromide (DDAB; 98%) and didodecyldimethyl ammonium chloride (DDAC; 98%) were purchased from Tokyo Chemical Industry. PEDOT:PSS (VP AI 4083) was purchased from Clevios. Poly-TPD and CBP were purchased from OSM. TPBi, 2,4,6-tris(3-(diphenylphosphinyl)phenyl)-1,3,5-triazine (PO-T2T), and poly(9-vinylcarbazole) were purchased from Lumtec. Poly(9,9-dioctylfluorene-*alt*-*N*-(4-*sec*-butylphenyl)-diphenylamine) was purchased from Solaris Technology. PDMS was purchased from Dow Corning.

### Synthesis of CsPbX_3_ PeNCs

CsPbX_3_ PeNCs were prepared by the reaction between cesium oleate and PbX_2_ (*54*, *59*). For a typical synthesis of green-emitting CsPbBr_3_ PeNCs, the cesium oleate solution was prepared by reacting Cs_2_CO_3_ (0.271 g) and OA (0.83 ml) in 1-ODE (10 ml) under vacuum at 120°C for 1 hour. In the other flask, the mixture of PbBr_2_ (0.138 g), 1-ODE (10 ml), OA (1.0 ml), and OAm (1.0 ml) was dried under vacuum at 120°C for 1 hour and then heated to 180°C under Ar atmosphere. At this temperature, 0.8 ml of cesium oleate solution was quickly injected into the PbBr_2_ solution. After 5 s, the reaction was quenched by the ice water bath. The products were purified by the standard centrifugation using acetonitrile and redispersed in cyclohexane for further uses.

Pristine PeNCs were passivated by OAm and OA. Because weak surface passivation of OAm ligands can result in the defect formation and/or degradation of PeNCs, we replaced OAm with halide-ion-pair ligands to improve optical properties and chemical stability of PeNCs (figs. S4 to S8) (*25*, *54*, *60*). For a typical ligand exchange process, 1.25 ml of DDAB solution (0.05 M in toluene) and 62.5 μl of OA were introduced into 8.0 ml of as-synthesized CsPbBr_3_ PeNC solution (6.25 mg ml^−1^ in toluene). The products were purified by the standard centrifugation using acetonitrile and redispersed in cyclohexane for further uses. Unless otherwise specified, the data in this study were collected using ligand-exchanged (i.e., DDAB/OA passivated) green CsPbBr_3_ PeNCs.

PeNCs with different colors (i.e., red and blue) were prepared by controlling the composition of halide contents of CsPbX_3_ PeNCs. For example, blue-emitting CsPb(Br_1-*x*_Cl*_x_*)_3_ PeNCs (*x* = 0.4) were prepared by the similar method for the preparation of green-emitting CsPbBr_3_ PeNCs except that DDAC was used instead of DDAB for the ligand exchange reaction. Red-emitting CsPbI_3_ PeNCs were prepared by the reaction between cesium oleate and PbI_2_.

### Double-layer transfer printing of PeNC patterns

The viscoelastic stamp for the pickup PeNC layer was fabricated with PDMS (Young’s modulus ~1 MPa). The PeNC layer was spin-cast on the ODTS-treated Si substrate (donor substrate) in the N_2_ glove box (*40*). A 25-nm-thick TPBi layer was thermally deposited on the spin-coated PeNC layer. Other organic layers (e.g., CBP or PO-T2T) can be used instead of TPBi. In the pickup step, the PDMS elastomeric stamp was contacted conformally on the PeNC/TPBi layer to prevent layer strain, pressed with the pressure of 196 kPa (~2 kgf cm^−2^), and then quickly detached from the donor substrate to pick up the PeNC/TPBi layer. For intaglio trench fabrication, the procedures were followed from previous research (*41*). The PeNC/TPBi double layer on the PDMS stamp was gently contacted to the intaglio trench. Because of the differences in surface energy between stamp and the intaglio trench, the contacted PeNC layer was transferred to the intaglio trench and desired patterns remained on the stamp. In the release step, the patterned PeNC/TPBi layer was released onto the receiver substrate (e.g., glass, Si wafer, or polyethylene terephthalate substrates). The laboratory-made aligner was used for the accurate subpixel alignment of different colored PeNC layers. As align keys were included in the pixelated monochromic PeNC layer, it was possible to precisely align the positions of the pixels and achieve multicolor patterned images. For the solvent treatment, a trace amount of methyl acetate was spin-cast to remove the organic layer and enhance the interfacial characteristics with the underlying layers. Unless otherwise specified, the data in this study were collected after the solvent treatment.

### FEM simulation

FEM simulations were used to analyze the stress distribution during the transfer printing of PeNCs. The simulations were performed for the release step (releasing patterns from the stamp to the receiver substrate). The substrate was modeled as a rigid body, whereas the structured stamp, the PeNC layer, and the TPBi layer were modeled using solid elements. The thickness of the PeNC layer is 13 nm and that of the TPBi layer is 25 nm. The incompressible Neo-Hookean model was used to represent the PDMS stamp.

### Fabrication of transfer-printed PeLEDs

To fabricate transfer-printed PeLEDs, patterned ITO glass was cleaned using ultrasonication process in deionized water and isopropyl alcohol. After 10 min of the UV ozone (UV/O_3_) treatment, the PEDOT:PSS layer was spin-coated at 5000 rpm for 40 s and annealed at 140°C for 10 min. The samples were transferred to a glove box, and poly-TPD (4 mg ml^−1^) HTL was deposited by spin-coating at 3000 rpm for 40 s and then annealed at 100°C for 3 min. The double-layered PeNC emitter with 25-nm-thick TPBi was transfer-printed from the donor substrate to the as-prepared poly-TPD film. The transfer printed TPBi layer was removed with methyl acetate, and the 50-nm-thick TPBi layer was additionally deposited by thermal evaporation. Last, LiF/Al (1.5 nm/90 nm) electrodes were thermally evaporated under high-vacuum condition.

### Fabrication of HODs and EODs

For fabrication of HODs, ITO/PEDOT:PSS/poly-TPD/PeNCs layers were deposited using the same procedures as PeLED fabrications. After coating processed PeNC layers, CBP, MoO*_x_*, and Au were deposited through thermal evaporation.

For fabrication of EODs, SnO_2_ was spin-coated on UV/O_3_-treated ITO substrates at 4000 rpm. After annealing at 140°C for 30 min in air, PeNCs/TPBi/LiF/Al layers were deposited using the same procedures as PeLED fabrications.

### Fabrication of transfer-printed wearable PeLEDs

The nickel sacrificial layer was thermally evaporated on the Teflon-treated glass substrate. Parylene-C (1 μm thick) was deposited on the Ni-coated glass substrate, followed by spin-coating of a 600-nm-thick epoxy layer (MicroChem). For device fabrication, ITO was sputtered with desired patterned metal mask on the substrate (55 W, 60 min, 3 mtorr, 80°C). Filtered PEDOT:PSS was spin-coated on the bottom electrode with 5000 rpm after the O_2_ plasma treatment, and the resulting layer was annealed for 20 min at 120°C. Later procedures were conducted in a glove box to hinder the degradation of PeNCs. Poly-TPD (4 mg ml^−1^) was spin-coated and annealed at 100°C for 3 min. Then, the patterned PeNC layer was transfer-printed with elastomer stamp on the HTL. The TPBi layer was thermally evaporated with 50-nm thickness using thermal evaporator after the solvent treatment to enhance the junction characteristics. Last, the anode, LiF/Al (1.5 nm/90 nm), was deposited to complete the device fabrication, and Parylene-C was deposited for device protection. Fabricated device was detached from the glass substrate, and the Ni sacrificial layer was dissolved in the Ni etchant.

### Characterization

Absorption spectra of PeNCs were recorded with a Cary 5000 UV-Vis-NIR spectrophotometer (Agilent Technologies), and PL spectra were measured using a Fluoromax-4 spectrophotometer (Horiba). Time-resolved PL spectroscopy data of the PeNC films on Si wafers were obtained by a time-correlated single-photon-counting setup (FluoTime 300, PicoQuant). The excitation wavelength of the red, green, and blue PeNC layers is 510, 450, and 375 nm, respectively.

X-ray diffraction patterns were measured using a Miniflex 600 x-ray diffractometer (Horiba). Fourier transform infrared spectra were measured using Cary 660 (Agilent Technologies). TEM analysis of PeNCs was performed using a Tecnai G2 F20 Twin TMP microscope (FEI). Cross-sectional TEM images of PeLEDs were acquired using a transmission electron microscope with JEM-2100F and the focused ion beam (Helios NanoLab 450). X-ray photoelectron spectroscopy spectra were measured using an ESCALAB 250Xi analyzer (Thermo Fisher Scientific). The x-ray beam size was 900 μm^2^ with a depth of 10 nm.

The resolution of patterns was estimated as follows. For dot patterns, the density of pixels in the diagonal direction was calculated in terms of PPI. For RGB arrays, a pixel composed of RGB subpixels was used as a unit pixel, and the diagonal resolution was calculated in terms of PPI. For line patterns, line pairs per inch was calculated on the basis of the horizontal density of patterns. The interdot or interline distance was considered for the estimation.

The contact angle results were measured by the low-bond axisymmetric drop shape analysis method using polar (deionized water) and nonpolar (glycerol) solvents on the ODTS-treated Si substrate. The surface energy of the PeNC layer and the PeNC/TPBi double layer was calculated from obtained polar and dispersive components, respectively. The work of adhesion value between the PDMS stamp and each layer was calculated using each obtained component and Eq. 2

$$W_{1,2}=4(\frac{\gamma_{1}^{d}\gamma_{2}^{d}}{\gamma_{1}^{d}+\gamma_{2}^{d}}+\frac{\gamma_{1}^{p}\gamma_{2}^{p}}{\gamma_{1}^{p}+\gamma_{2}^{p}})$$ (2)

where *W*_1,2_ is the work of adhesion between materials 1 and 2, and γ is the component of the surface energy corresponding to each subscript.

The morphology of PeNC films was characterized with atomic force microscopy (Bruker Nano Surface, Dimension Icon), fluorescence confocal microscopy (Zeiss, LSM 980), and field-emission scanning electron microscopy (Nova Nano230). GI-SAXS analysis was performed at the PLS-II 3C and 6D UNIST-PAL beamline of the Pohang Accelerator Laboratory (PAL), Korea.

The device performances (EL spectra, *J-V-L* curves, EQE values, and CIE coordinates) of PeLEDs were measured from 0.0 to 6.0 V with a step voltage of 0.2 V through a Keithley 2400 source meter and a Konica Minolta CS-2000 spectrometer under the ambient air conditions. Electrochemical impedance analysis data of PeLEDs were collected by a PGSTAT302N electrochemical workstation combined with the FRA32M module (Autolab).
