# Supplementary material for: Ultrahigh-resolution full-color perovskite nanocrystal patterning for ultrathin skin-attachable displays
Source: Sci Adv. 2022 Oct 26;8(43):eadd0697. doi: 10.1126/sciadv.add0697 (PMC9604611; doi:10.1126/sciadv.add0697)
Supplement: Supplementary file 1 — Figs. S1 to S27 Tables S1 to S4 [file sciadv.add0697_sm.pdf]

Science Advances
AAAS

Supplementary Materials for

Ultrahigh-resolution full-color perovskite nanocrystal patterning for ultrathin skin-attachable displays

Jong Ik Kwon *et al.*

Corresponding author: Moon Kee Choi, mkchoi@unist.ac.kr; Jiwoong Yang, jiwoongyang@dgist.ac.kr

Sci. Adv. **8**, eadd0697 (2022)
DOI: 10.1126/sciadv.add0697

This PDF file includes:

Figs. S1 to S27
Tables S1 to S4

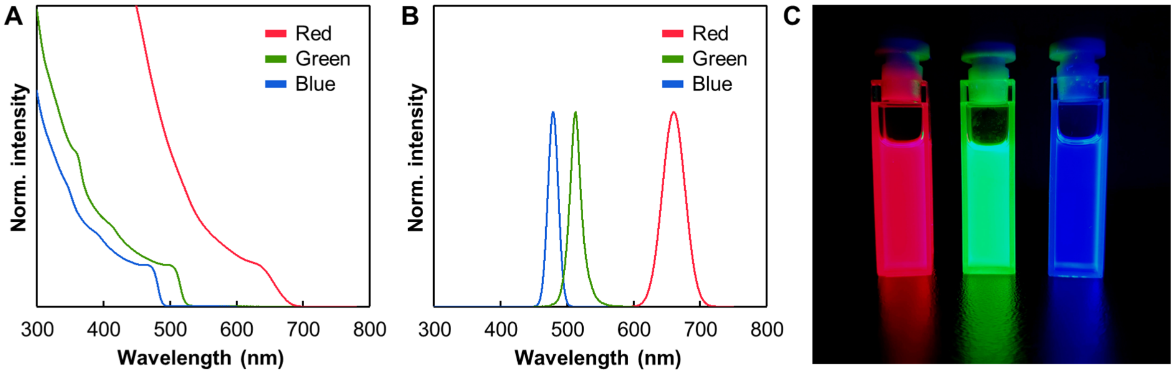

Fig. S1. Optical properties of $CsPbX_3$ perovskite nanocrystals (PeNCs). (**A**) Normalized absorption spectra, (**B**) normalized photoluminescence (PL) spectra, and (**C**) photograph (under UV irradiation, λ = 365 nm) of red, green, and blue PeNC solutions. All the data were collected after the ligand exchange process.

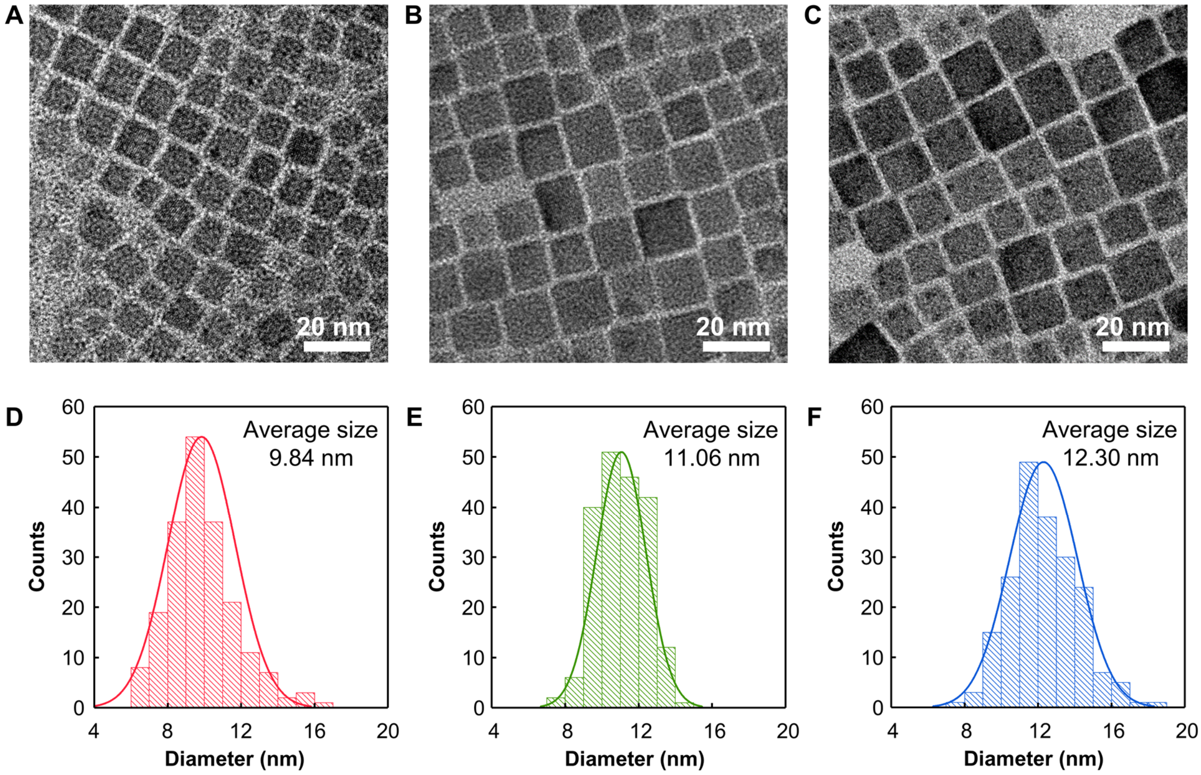

Fig. S2. Transmission electron microscopy (TEM) analysis of $CsPbX_3$ PeNCs. (**A** to **C**) TEM images of (A) red, (B) green, and (C) blue PeNCs. (**D** to **F**) Histograms (N = 200) showing the size distribution of (D) red, (E) green, and (F) blue PeNCs. Average sizes of red, green, and blue PeNCs are 9.84 nm (±1.83 nm), 11.06 nm (±1.34 nm), and 12.30 nm (±1.85 nm), respectively. All the data were collected after the ligand exchange process.

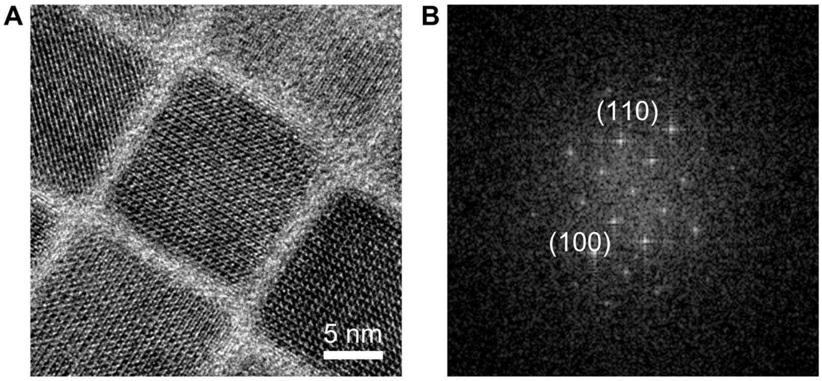

Fig. S3. High-resolution TEM analysis. (**A**) High-resolution TEM image and (**B**) the corresponding fast Fourier transform (FFT) pattern of ligand exchanged green PeNCs.

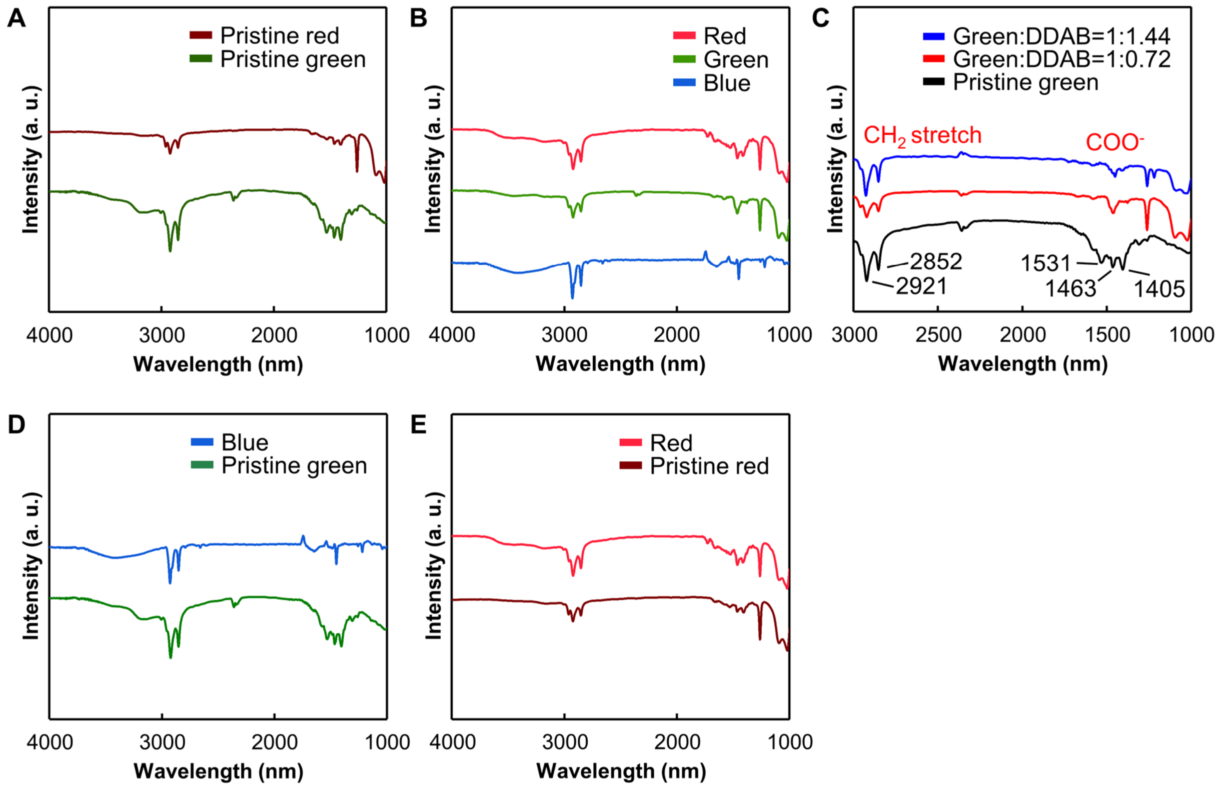

Fig. S4. Fourier transform infrared (FTIR) spectroscopy of $CsPbX_3$ PeNCs. (**A**) FT-IR spectra of red and green PeNCs before the ligand exchange process. (**B**) FT-IR spectra of red, green, and blue PeNCs after the ligand exchange process. (**C**) The change of FT-IR spectra of green PeNCs depending on the relative molar ratio of PeNCs:DDAB. The spectrum of green PeNCs (black solid line) before the ligand exchange process is presented together for comparison. (**D**) Comparison of FT-IR spectra of blue PeNCs before and after the ligand exchange process. (**E**) Comparison of FT-IR spectra of red PeNCs before and after the ligand exchange process.

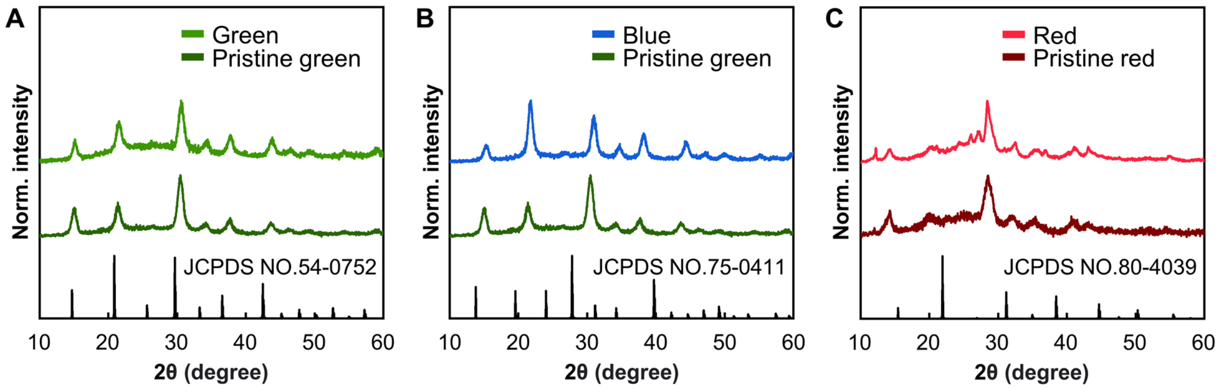

Fig. S5. X-ray diffraction (XRD) analysis of $CsPbX_3$ PeNCs. (**A** to **C**) Normalized XRD patterns of (A) green, (B) blue, and (C) red PeNCs before and after the ligand exchange process. The reference XRD patterns are shown together for comparison. The reference data are acquired from bulk crystal structures of cubic ($CsPbBr_3$: JCPDS No. 54-0752, $CsPbCl_3$: JCPDS No. 75-0411, and $CsPbI_3$: JCPDS No. 80-4039) phases.

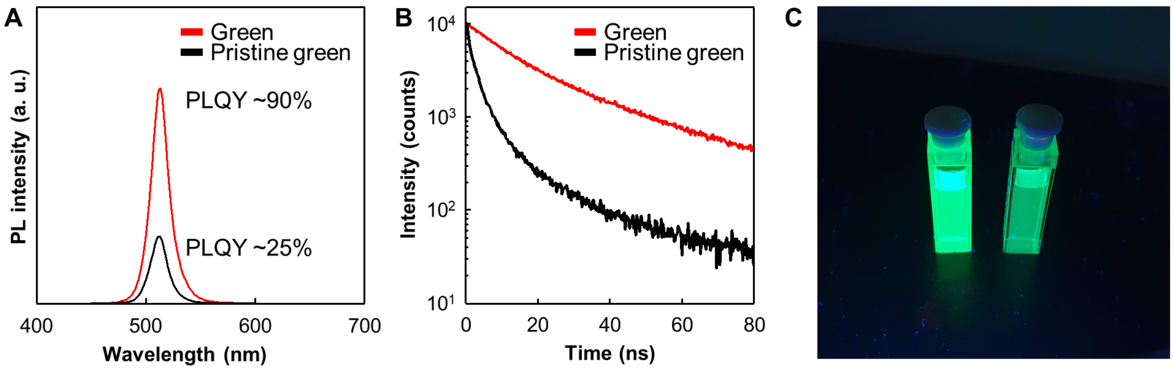

Fig. S6. Comparison of optical characteristics of pristine and ligand-exchanged PeNCs. (**A** and **B**) (A) Steady-state PL and (B) time-resolved photoluminescence (TRPL) spectra of pristine (black) and ligand-exchanged green PeNCs (red) dispersed in cyclohexane. (**C**) Photograph (under UV irradiation, λ = 365 nm) of green PeNC colloidal solutions before (right) and after (left) the ligand exchange.

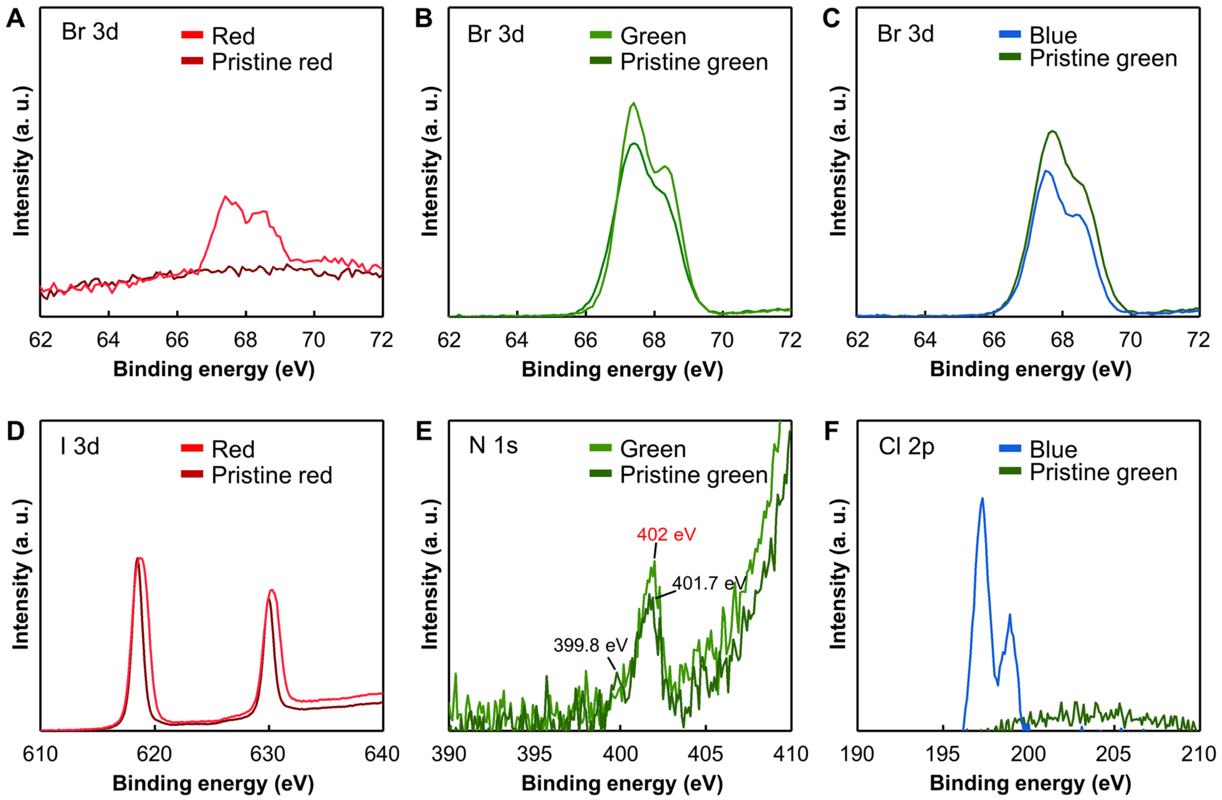

Fig. S7. X-ray photoelectron spectroscopy (XPS) analysis of $CsPbX_3$ PeNCs. (**A** to **C**) Br 3d spectra of (A) red, (B) green, and (C) blue PeNCs. (**D**) I 3d spectra of red PeNCs before and after the ligand exchange process. (**E**) N 1s spectra of green PeNCs before and after the ligand exchange process. (**F**) Cl 2p spectra of blue PeNCs before and after the ligand exchange process.

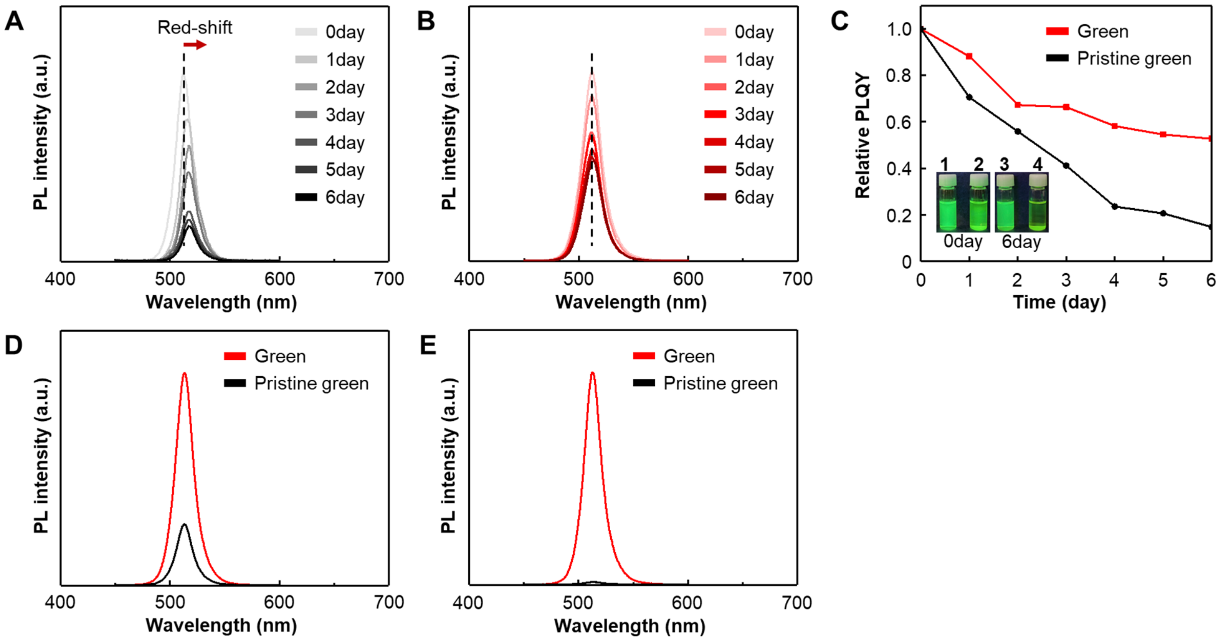

Fig. S8. Stability test results of pristine and ligand-exchanged PeNCs. (**A** and **B**) PL spectra acquired during the stability test of (A) pristine and (B) ligand-exchanged PeNCs dispersed in cyclohexane. The solutions were exposed to the ambient air for 6 days (25 ºC, relative humidity: ~50%). (**C**) Relative PLQY of green PeNCs before (black) and after (red) ligand exchange as a function of the time under the ambient air condition. Insets show photographs of green PeNC solutions before (left) and after (right) the air exposure: sample 1 and 3 are ligand exchanged green PeNCs, sample 2 and 4 are pristine green PeNCs. (**D** and **E**) PL spectra of the green PeNC layers using pristine PeNCs and ligand-exchanged PeNCs (D) before and (E) after the exposure to the polar solvent (methyl acetate was spin-cast on PeNC layers).

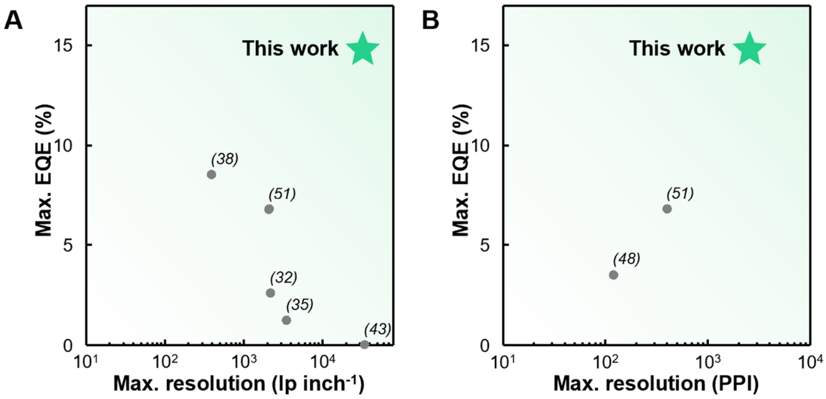

Fig. S9. Comparison of key characteristics (maximum resolution and maximum EQE) of the printed perovskites. (**A** and **B**) Comparison of key characteristics of printed (A) line pattens and (B) aligned RGB patterns.

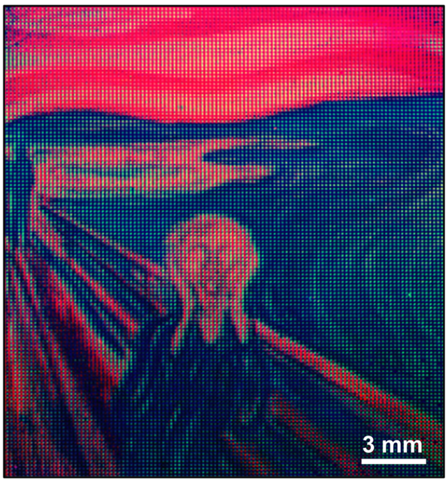

Fig. S10. PL image of transfer-printed red/green/blue (RGB) PeNC pixelated patterns displaying *The Scream* by Edvard Munch.

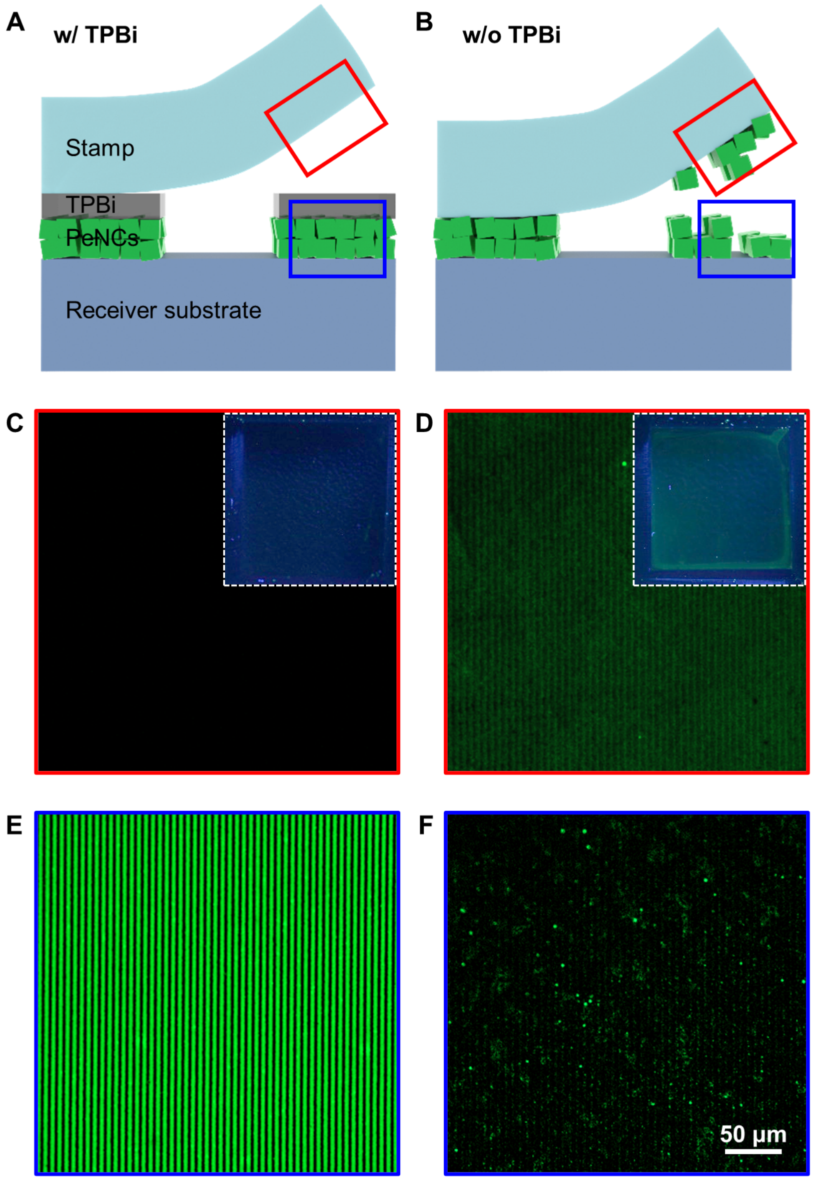

Fig. S11. Effect of the organic layer for dry transfer printing. (**A** and **B**) Schematic illustration showing the patterned PeNC films (A) with and (B) without TPBi layer at the release step in the double-layer transfer printing process. (**C** and **D**) Fluorescence microscopic images of stamps after the release step (C) with and (D) without TPBi layer. The insets show PL images of the stamps. (**E** and **F**) Fluorescence microscopic images of PeNC patterns printed (E) with and (F) without TPBi layer.

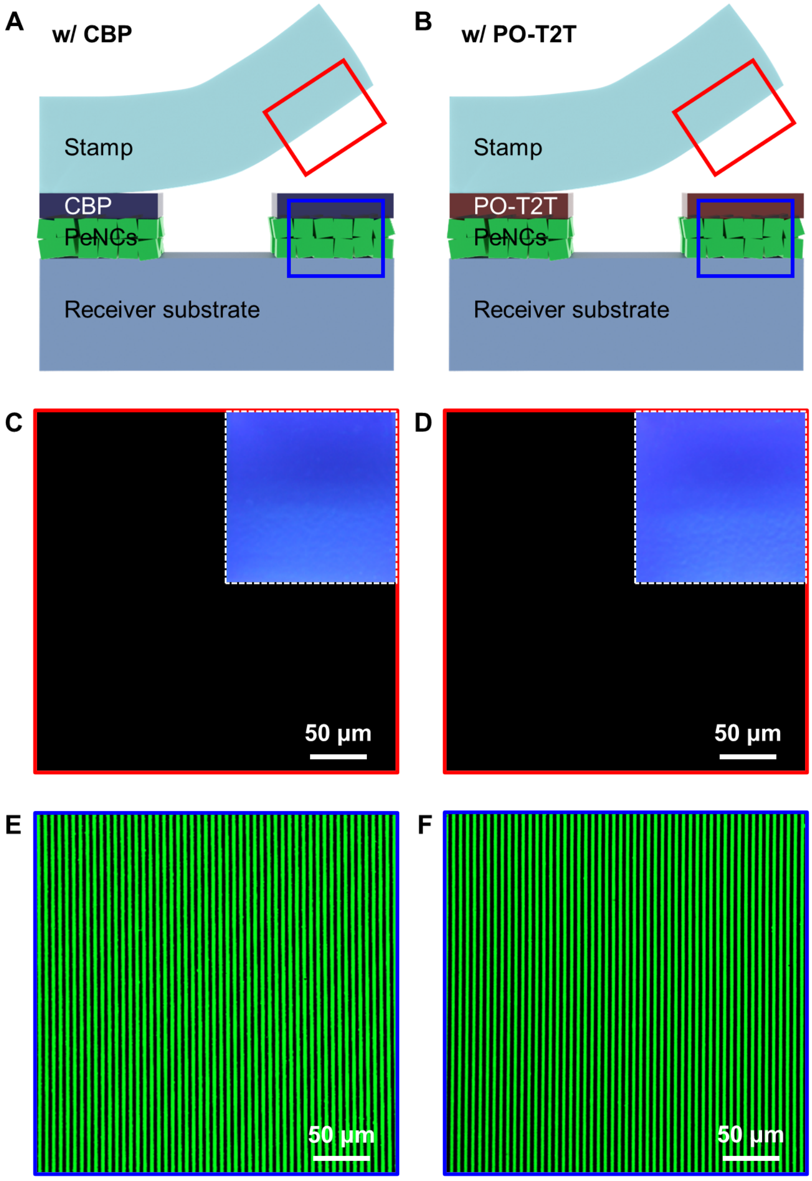

Fig. S12. Effect of the various organic layers for the double-layer transfer printing. (**A** and **B**) Schematic illustration showing the patterned PeNC films using (A) CBP and (B) PO-T2T for organic layers the release step in the double-layer transfer printing process. (**C** and **D**) Fluorescence microscopic images of stamps after the release step with (C) CBP and (D) PO-T2T organic layers. The insets show PL images of the stamps. (**E** and **F**) Fluorescence microscopic images of PeNC patterns printed with (E) CBP and (F) PO-T2T organic layer.

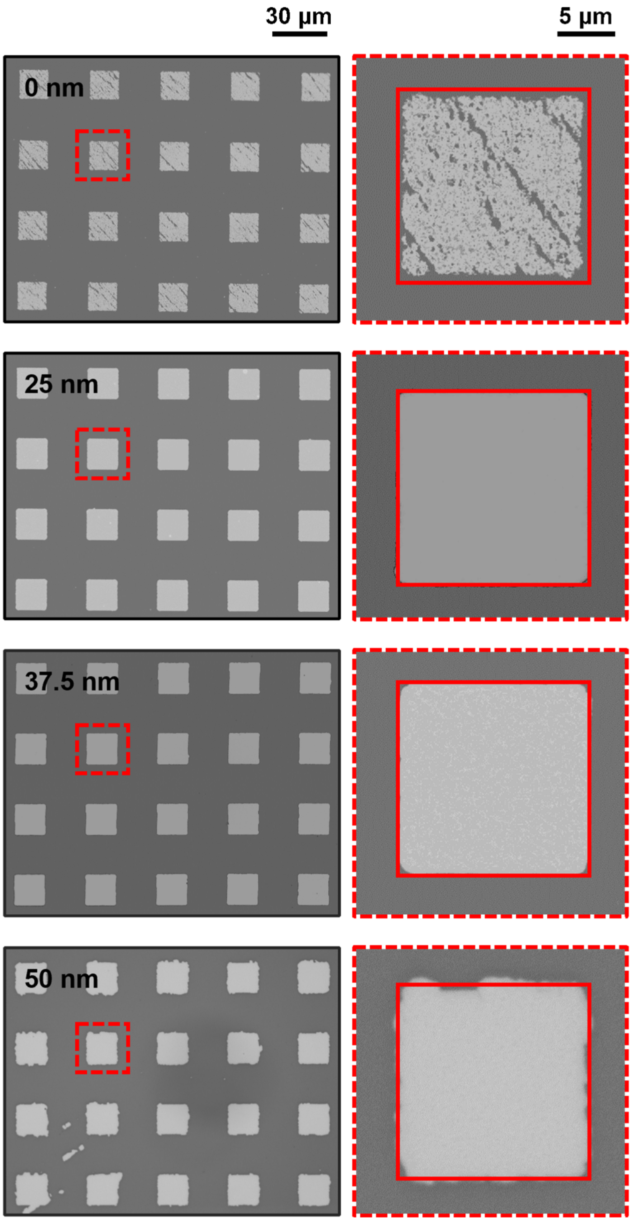

Fig. S13. Optical images of transfer-printed PeNCs with 0 nm, 25 nm, 37.5 nm, and 50 nm TPBi layers.

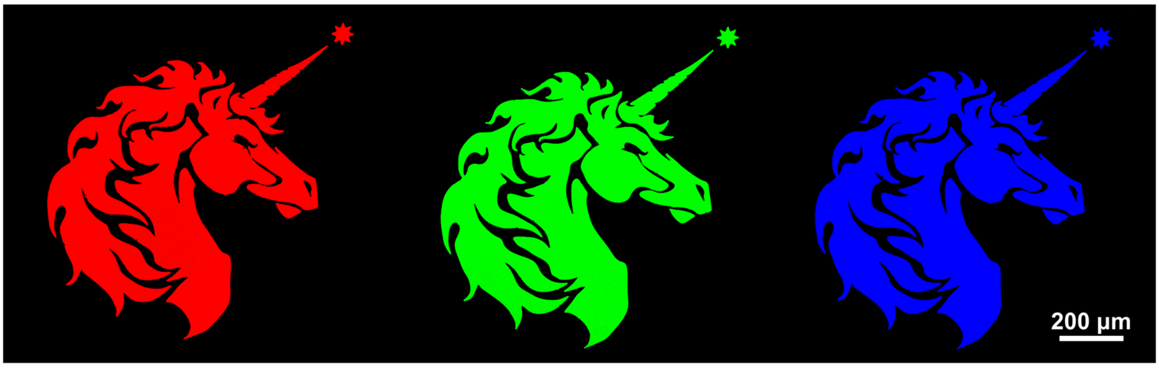

Fig. S14. Fluorescence microscopic images of red, green, and blue PeNC patterns with complex shape.

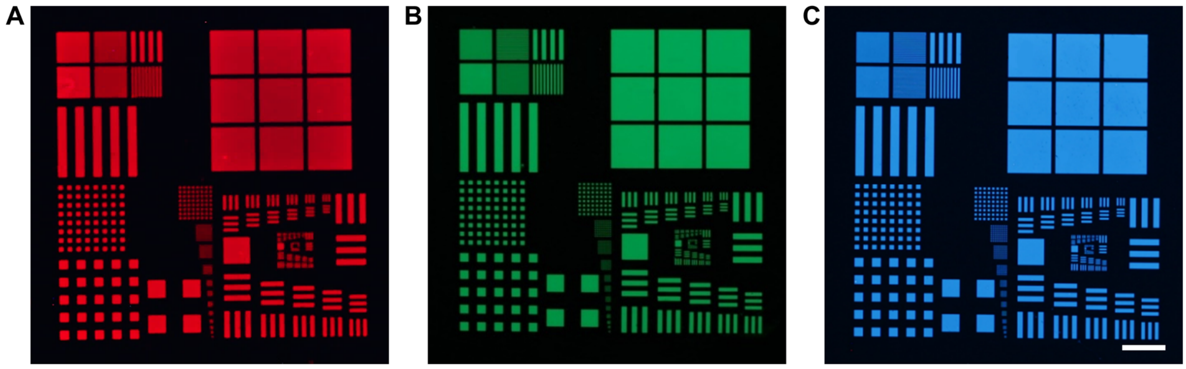

Fig. S15. PL images of transfer-printed PeNC patterns with various size and shape. (**A** to **C**) Patterns with (A) red, (B) green, and (C) blue PeNCs (scale bar: 1 mm).

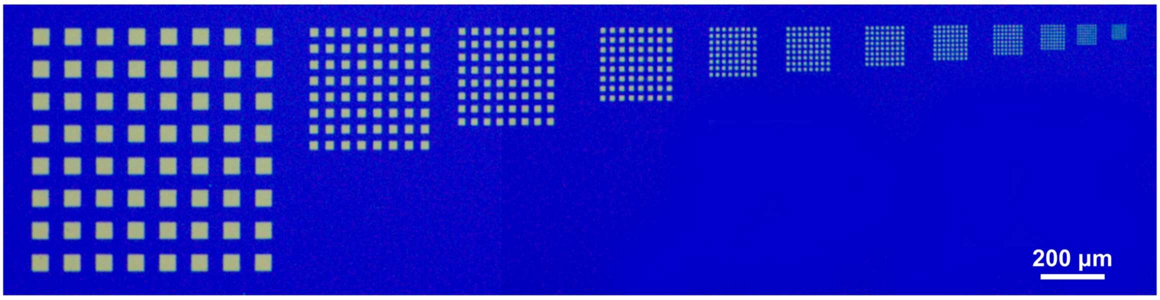

Fig. S16. Optical image showing a series of pixelated PeNC arrays with a wide range of pixel sizes (from 3 μm to 50 μm).

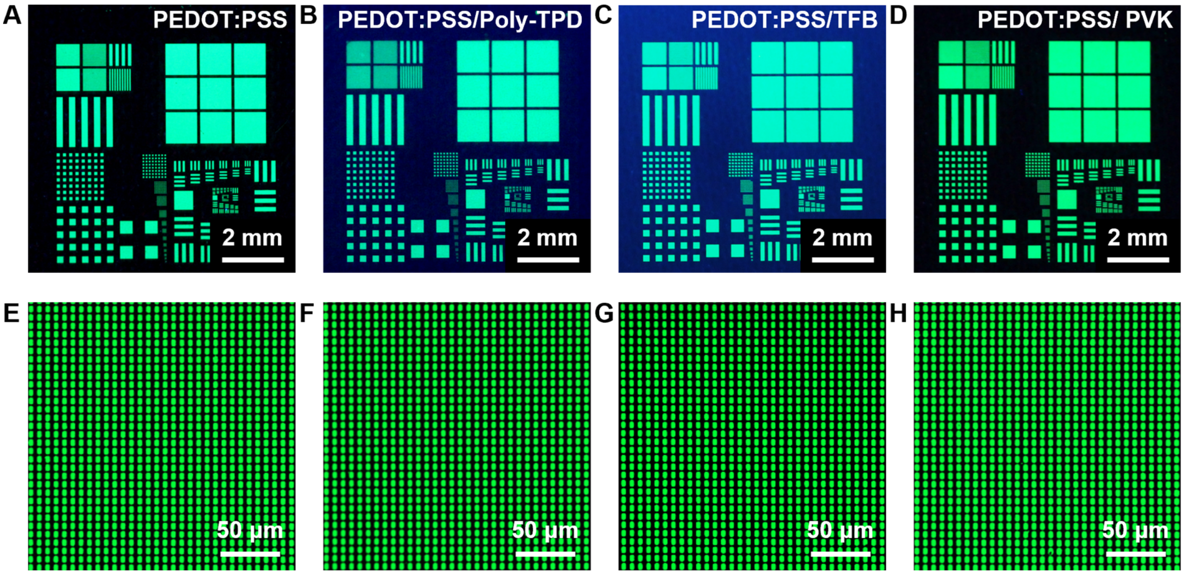

Fig. S17. PL images of double-layer transfer-printed PeNC patterns on various charge transport layers. (**A** to **D**) PL images of transfer-printed multiscale PeNC patterns onto the various charge transport layers, including (A) PEDOT:PSS, (B) PEDOT:PSS/Poly-TPD, (C) PEDOT:PSS/TFB, and (D) PEDOT:PSS/PVK. (**E** to **H**) Fluorescence microscopic images of high-resolution transfer-printed PeNC pixel arrays (width: 3 μm) onto the various charge transport layers, including (E) PEDOT:PSS, (F) PEDOT:PSS/Poly-TPD, (G) PEDOT:PSS/TFB, and (H) PEDOT:PSS/PVK.

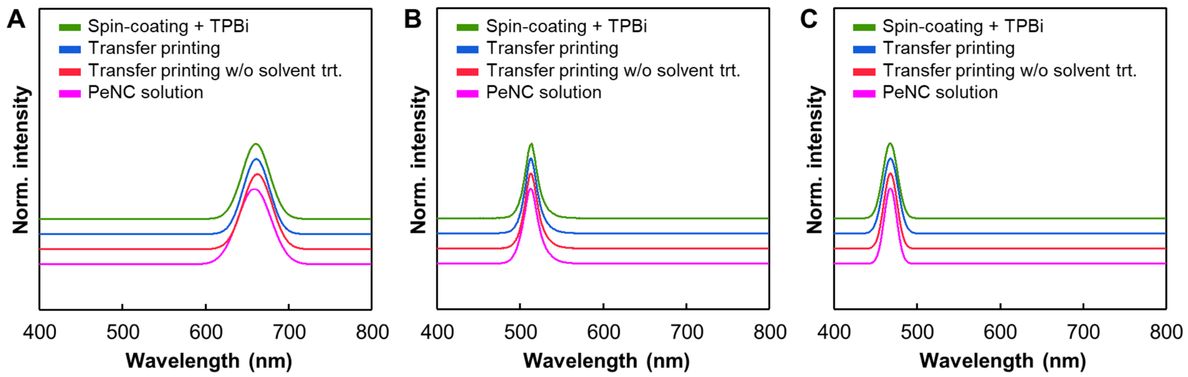

Fig. S18. PL analysis of PeNC films with various film formation methods. (**A** to **C**) PL spectra of (A) red, (B) green, and (C) blue PeNC films with various film formation methods (spin-coating + TPBi, transfer printing, and transfer printing without solvent treatment). The spectra of PeNC solutions are presented together for comparison.

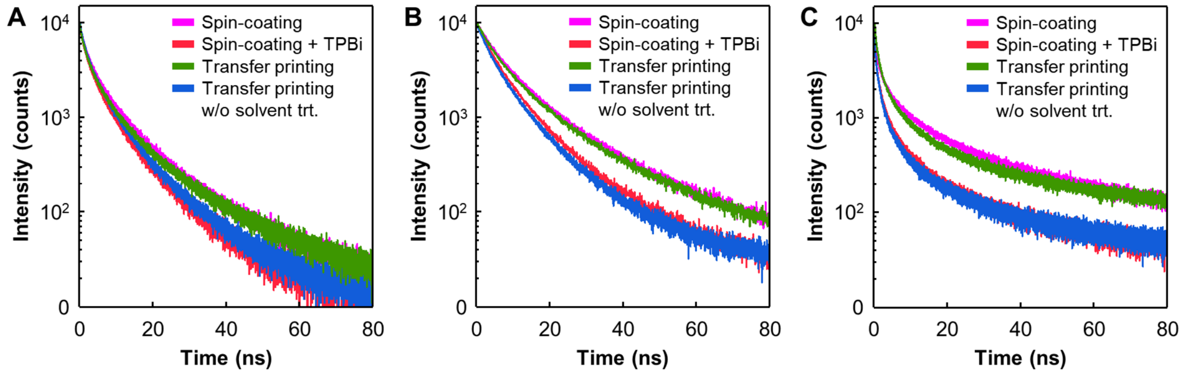

Fig. S19. Time-resolved PL analysis of PeNC films with various film formation methods. (**A** to **C**) TRPL spectra of (A) red, (B) green, and (C) blue PeNCs with various film formation methods (spin-coating, spin-coating + TPBi, transfer printing, and transfer printing without solvent treatment).

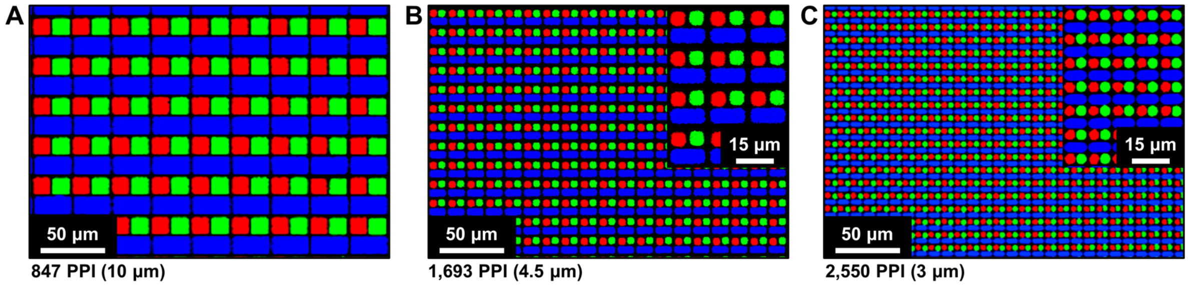

Fig. S20. Fluorescence microscopic images of the pixelated RGB PeNC patterns. (**A** to **C**) Fluorescence microscopic images of the pixelated RGB PeNC patterns with (A) 847, (B) 1,693, and (C) 2,550 PPI resolutions.

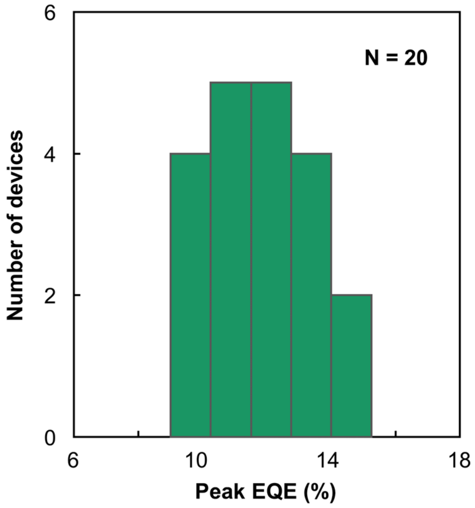

Fig. S21. Histogram of peak EQE of transfer-printed PeLEDs (N = 20).

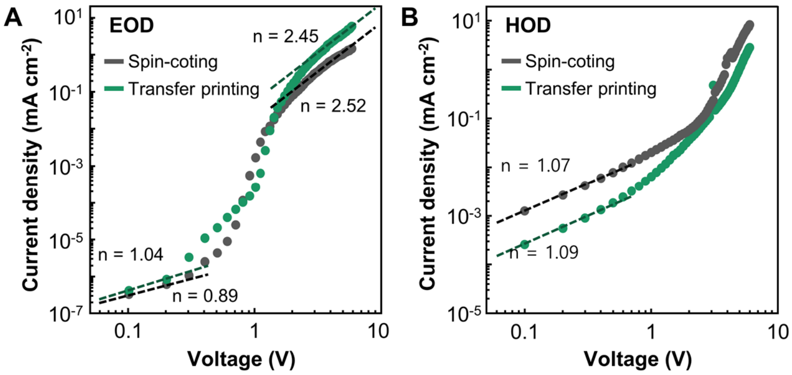

Fig. S22. Analysis of electron-only and hole-only devices. (**A** and **B**) $J-V$ curves of (A) electron-only and (B) hole-only devices with different PeNC film formation methods.

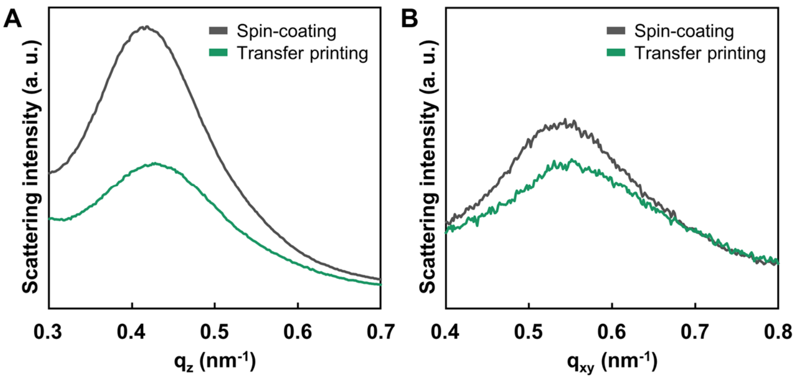

Fig. S23. Grazing incidence small-angle X-ray scattering (GI-SAXS) analysis of PeNC films. (**A** and **B**) GI-SAXS data of PeNC films according to fabrication processes in (A) out-of-plane and (B) in-plane direction.

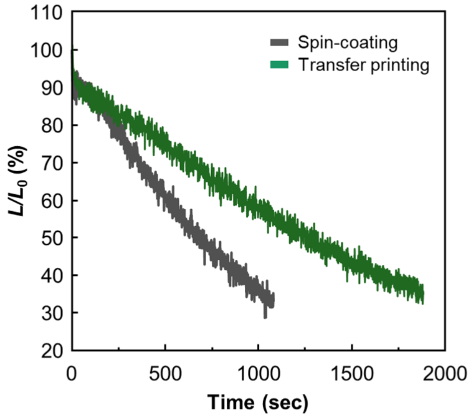

Fig. S24. The device lifetimes of PeLEDs with different PeNC film formation methods at an initial luminance (L_0) of 100 cd m^{-2}.

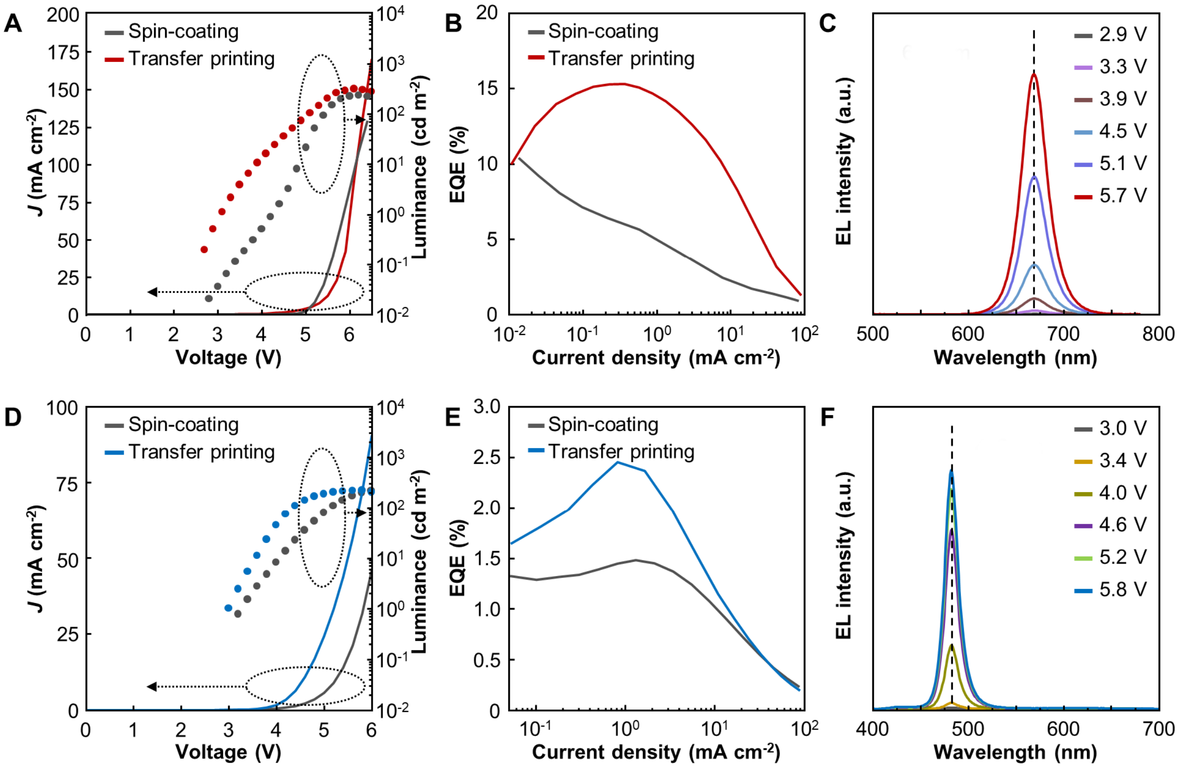

Fig. S25. EL characteristics of transfer-printed red and blue PeLEDs. (**A**) *J*−*V*−*L* curves and (**B**) EQE versus current density of the PeLEDs using different film formation methods with red PeNCs. (**C**) EL spectra of the transfer-printed red PeLEDs at various applied biases. (**D**) *J*−*V*−*L* curves and (**E**) EQE versus current density of the PeLEDs using different film formation methods with blue PeNCs. (**F**) EL spectra of the transfer-printed blue PeLEDs at various applied biases.

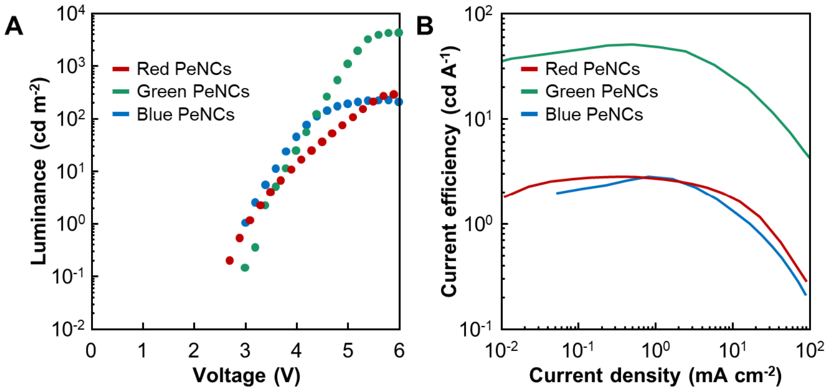

Fig. S26. EL Characteristics of transfer-printed RGB PeLEDs. (**A**) Luminance and (**B**) current efficiency versus current density of transfer-printed RGB PeLEDs.

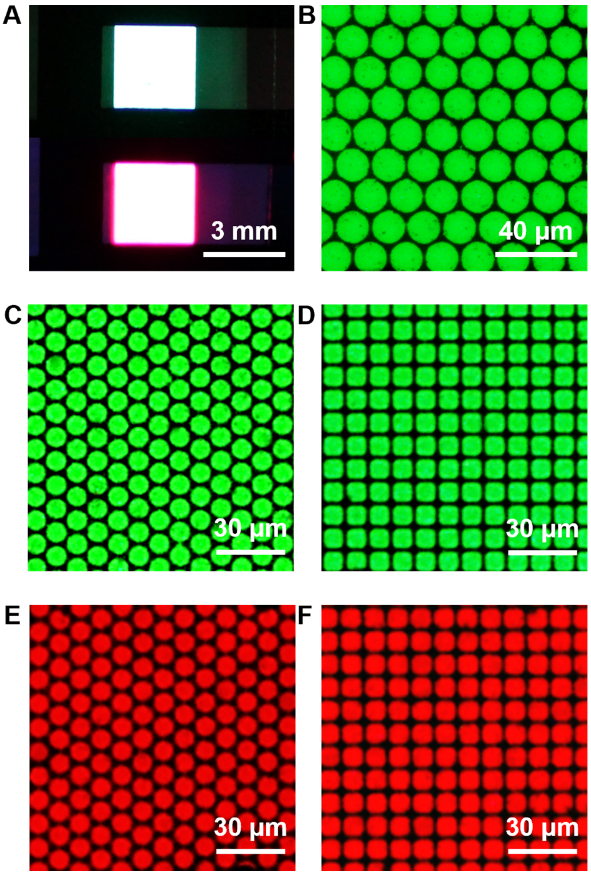

Fig. S27. **Optical microscopy images of the EL emission from high-resolution transfer-printed PeLEDs.** (**A**) Photograph of transfer-printed PeLEDs with high-resolution green and red PeNC patterns. (**B** to **D**) Optical microscopy images of the EL emission from high-resolution transfer-printed green PeLEDs; (B) 16 μm circle, (C) 8 μm circle, and (D) 7 μm (2,550 PPI) square patterns. (**E** and **F**) Optical microscopy images of the EL emission from high-resolution transfer-printed red PeLEDs; (E) 8 μm circle and (F) 7 μm (2,550 PPI) square patterns.

Table S1. The summary of characteristics of printed perovskites.

Fabrication method	Dot pattern width	Line pattern width	RGB subpixel width	Mate rial	Max. efficiency	Ref.
Transfer printing	800 nm (16,000 PPI)	400 nm (33,000 line pairs/inch)	3 µm × 3 µm (2,550 PPI)	NC	R: 15.3%<br>G: 14.8%<br>B: 2.5%	This work
Photolithograph y (ligand crosslinking)	~1 µm	~1 µm		NC	G: 6.7 cd/A at 100 cd/m^2	(*32*)
Photolithograph y (lift-off)	10 µm	4 µm		NC	G: 1.24%	(*35*)
Photolithograph y (ligand crosslinking)	5 µm × 14 µm	12 µm	15 µm × 60 µm	NC	G: 6.8%	(*51*)
Inkjet printing	~70 µm	~70 µm		NC	R: 5.54%<br>G: 8.54%<br>B: 0.81%	(*38*)
Inkjet printing	50 µm × 190 µm			NC	G: 2.8%	(*46*)
Inkjet printing	120 µm × 300 µm			NC	G: 3.03%	(*47*)
Inkjet printing	~70 µm		50 µm × 190 µm (120 PPI)	Quas i-2D	R: 3.5%<br>G: 3.4%<br>B: 1.0%	(*48*)
Inkjet printing	30 µm			Quas i-2D	G: 9.0%	(*49*)
Nanostructure templates		~500 nm		NC	G: 0.012%	(*43*)
Homopitaxial growth	20 µm			Bulk	G: 6.1%	(*44*)
Screen printing	20 µm			Bulk	G: < 0.1%	(*45*)
Thermal evaporating	~100 µm			NC	G: 8%	(50)

Table S2. Work of adhesion between PeNC layers and a PDMS stamp.

Layer	Contact angle [°][a)]		γ^{d} (Dispersion components) [mJ/m^2][b)]	γ^{p} (Polar components) [mJ/m^2][c)]	Work of adhesion [mJ/m^2][d)]
	Water	Glycerol			
**PeNC**	94.23	88.33	9.44	7.35	28.11
**PeNC/TPBi**	98.60	100.76	1.66	12.19	9.11
**PDMS**	-	-	19.0	0.8	-

[a)] After each layer deposited on the silicon wafer, constant volume of droplet was slowly contact with the sample.

[b,c)] Dispersion and polar components are calculated by the Fowkes model using glycerol and deionized water.

[d)] Work of adhesion between the PDMS stamp and each layer.

$$W_{1,2} = 4\left(\frac{\gamma_1^d \gamma_2^d}{\gamma_1^d + \gamma_2^d} + \frac{\gamma_1^p \gamma_2^p}{\gamma_1^p + \gamma_2^p}\right)$$

Table S3. Electrochemical impedance analysis. The data were aquired using the equivalent circuit in Fig. 3F (applied bias = 4.0 V).

**Process**	**R_s [Ω]**	**R_{ct} [Ω]**
Spin-coating	67.09	2.23×10^{4}
Transfer printing	39.80	2.95×10^{3}
Transfer printing without solvent treatment	119.96	1.57×10^{6}

Table S4. GI-SAXS data of PeNC films with different film formation methods.

Process	Out-of-plane		In-plane	
	θ [degree]	Distance [nm]	θ [degree]	Distance [nm]
Spin-coating	0.0417	15.07	0.0544	11.55
Transfer printing	0.0429	14.65	0.0551	11.40
